# Can we microbe-manage our vitamin acquisition for better health?

**DOI:** 10.1371/journal.ppat.1011361

**Published:** 2023-05-18

**Authors:** Jana Nysten, Patrick Van Dijck

**Affiliations:** Laboratory of Molecular Cell Biology, Department of Biology, Institute of Botany and Microbiology, KU Leuven, Leuven, Belgium; Duke University School of Medicine, UNITED STATES

## Introduction

Vitamins are indispensable micronutrients that are needed for a myriad of metabolic and regulatory processes critical for all living organisms [1–3]. Because humans do not have the capacity to synthesize most of these essential nutrients themselves, they need to be obtained externally. Even though vitamins can be found in various foods, deficiencies still occur in developing and industrialized countries [4]. A rather unexpected source of vitamins is the microbiota present in the gastrointestinal (GI) tract. Most of these microbes can synthesize vitamins de novo and even produce them in excess, notably vitamin K and vitamins in the B group such as riboflavin, niacin, and cobalamin [5,6]. It has been estimated that they produce up to 30% of the recommended daily intake for the host; however, this depends on the microbiome composition and the host’s diet [7]. Moreover, microbes can work together to produce vitamins that can modulate the metabolic activity and composition of the human gut microbiome [8,9].

Interestingly, recent research has highlighted the importance of vitamins in the pathology of opportunistic pathogens that live as commensals in the human GI tract [10,11].

## The advantages of vitamin production by gut microbiota

**Fig 1:**
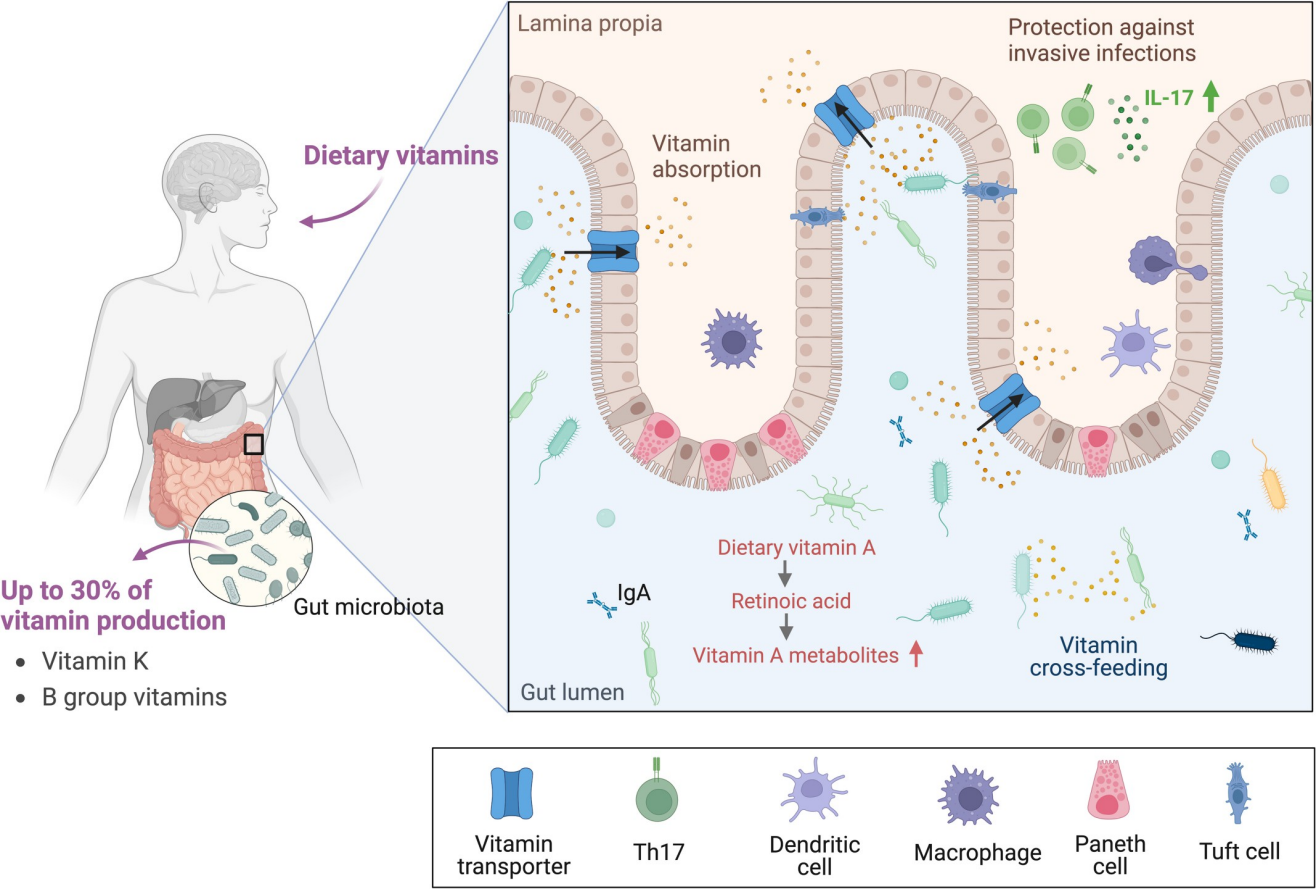
The role of gut microbiota in vitamin production. Humans acquire vitamins through their diet and the microbiota in the large intestine, which can produce up to 30% of the recommended daily intake of vitamin K and the B group vitamins. The excreted vitamins can be used to cross-feed other species or can be absorbed by the host. Besides vitamin secretion, commensals in the GI tract can convert dietary vitamin A into active vitamin A metabolites, which are important for maintaining epithelial homeostasis and immune function. Created with BioRender.com. GI, gastrointestinal; IgA, immunoglobulin A; IL-17, interleukin 17; Th17, T-helper 17.

The human GI tract harbors a large and complex population of microorganisms that offers a range of physiological functions and are consequently imperative for the host’s health [1,2]. It has been shown that vitamins can beneficially modulate the microbiota in the GI tract by expanding the abundance of commensals, enlarging the microbial diversity, altering the amount of short-chain fatty acids, or modulating the barrier function or immune response [9]. Most of the human GI tract’s commensals can biosynthesize and secrete vitamin K and B group vitamins, but besides excess vitamin biosynthesis, the gut microbiota can also convert dietary vitamin A precursors into retinoic acid, a key regulator of gene expression [12,13]. This elevates the concentration of vitamin A metabolites, aids in maintaining immune homeostasis, and helps to prevent pathogen invasion (Fig 1) [13]. Furthermore, vitamins with antioxidant properties can affect infectious diseases, either directly or indirectly by tuning the immune system or the redox state [14,15].

## Vitamin cross-feeding: Teamwork makes the dream work

The microbial community of the GI tract relies on dynamic interactions among microbes that sustain a complex network of interconnected metabolisms [16]. Hundreds of metabolites are consumed, secreted, and shared in a phenomenon called cross-feeding, which leads to interspecies interactions, which can directly impact the human host [17,18].

According to genome annotation studies, 40% to 65% of human gut commensals produce at least one of the 8 vitamins belonging to the B group. While some organisms produced all 8, some had no de novo vitamin synthesis, which led to an interesting observation where pairs of organisms have complementary vitamin synthesis pathways [8]. This indicates that the gut microbes actively exchange vitamins among each other, suggesting that cooperation between species is necessary and may have led to the coevolution of certain species (Fig 1) [8]. An interesting example is vitamin B_12_ or cobalamin, a rather unusual vitamin as it is exclusively synthesized by microorganisms. The de novo synthesis is complicated and energetically demanding as it takes 30 enzymatic steps [19]. Therefore, some species salvage intermediates of the synthesis or the final cobalamin product from other species [20]. It has been shown that *Clostridioides difficile*, an opportunistic pathogen and common inhabitant of the human gut, down-regulates its cobalamin biosynthesis in the presence of *Clostridium sardiniense*. This indicates the cross-feeding of vitamin B_12_, which consequently influences community population dynamics. Interestingly, the combination of these organisms worsens the pathogen’s virulence *in vivo* partly by adapting the metabolism to one another, showing that commensals can alter the GI tract nutrient environment to modulate the virulence of pathogens [21].

## The benefits of harboring opportunistic pathogens in the GI tract

The human GI tract is home to a variety of opportunistic pathogens such as *Candida albicans* that can cause invasive diseases with extremely high morbidity and mortality [22]. However, these pathogens are present in the GI tract of most people without causing any infection. This commensal state is underexplored, and it remains unclear what these potential pathogens are doing in the healthy GI tract. It seems likely that an opportunistic pathogen in the human GI tract would elicit some host benefit as a solely harmful pathogen would be subjected to a negative selection [23]. These benefits may provide an evolutionary pressure for establishing and maintaining opportunistic pathogens in the human gut. Rather remarkable about some pathogens is their excessive vitamin production. *C*. *albicans*, for example, secretes high levels of riboflavin, but it remains unclear why, as the physiological role for the overproduction is unknown [24]. It is conceivable that the produced vitamin can be absorbed by the host or other commensals and consequently provides a benefit for the human host.

Besides vitamin secretion, other advantages of harboring potential pathogens in the GI tract have been discussed before. It has been shown that these pathogens can train the immune system by stimulating the responsiveness of neutrophils and by providing protection against invasive infections (Fig 1) [23,25,26]. It is not surprising that opportunistic pathogens would offer some host benefits as they have coevolved for many years to form intricate and complex relationships [27]. In fact, these benefits most likely provide an evolutionary pressure for the establishment and maintenance of opportunistic pathogens as commensals in the gut.

## Nurture your microbiota for more vitamins

Throughout the last century, mankind has raged a war against bacteria [28]. While antibiotic treatments help many people, major concerns are arising regarding drug resistance and the importance of the microbiome for human health. By eliminating part of our microbiota, we disarm our own defense lines and become more susceptible to other infections, e.g., fungal diseases. Recently, probiotics have become one of the most popular food supplements to the point where they have become a fast-growing multibillion-dollar industry [29]. Most probiotics contain species of the *Bifidobacterium*, *Lactobacillus*, or *Saccharomyces* genera, all capable of de novo synthesis of vitamins [30]. The elevated vitamin production of specific strains could be a novel promising application of probiotics as it can be helpful for both the host and other microbial species as the vitamin can be synthesized and delivered directly into the intestine [31,32]. This would be especially useful for water-soluble vitamins such as B vitamins, which cannot be stored in the body and consequently require a constant supply. Furthermore, it has been shown that a riboflavin-overproducing *Lactobacillus* strain can prevent mucositis, a mucosal inflammation and common side effect of cancer treatments [33].

Apart from probiotics, diets can also influence vitamin production. It has been shown that urinary riboflavin excretion was increased among people with higher carbohydrate and low-fat diets, suggesting that riboflavin secretion by the microbiota was enhanced in this condition [34].

Altogether, there are interesting applications for vitamin-overproducing strains as their antioxidant and anti-inflammatory properties can improve host health and the vitamin is protected from the harsh environment of the GI tract as it is secreted directly into the large intestine [31,33,35,36].

## Vitamin fortification, beneficial or detrimental to human health?

In recent decades, the dietary patterns in high-income countries have shifted towards a higher calorie density and reduced nutritional value, which can lead to insufficient intake of certain micronutrients [37]. One strategy to enhance nutrient ingestion without increasing caloric intake is the fortification of food with vitamins, which has led to substantial health benefits [38,39]. Besides fortified foods, vitamins are also obtained from non-fortified foods and supplements, but the benefits of the latter are still debated in scientific research. While some studies suggest that vitamin supplements can increase gut microbial diversity and offer protection against infectious diseases, other research has shown that taking vitamins without medical justification does not reduce the risk of cardiovascular diseases or cancer and can even be harmful [40–47]. Excessive intake of fat-soluble vitamins (A, D, E, and K) can be particularly detrimental as these vitamins accumulate in adipose tissue, leading to adverse health effects. In contrast, water-soluble vitamins rarely accumulate in the body and are excreted by the kidneys. However, oral vitamin supplements typically provide high doses that surpass the recommended daily amount, which can alter the competitive or syntrophic interactions between gut microbes [48]. Studies with mice have demonstrated that vitamin B_12_ supplements promote colonization and pathogenesis of *Citrobacter rodentium*, a mice-specific pathogen, by altering the activities of *Lachnospiraceae* species. These findings emphasize that oversupplementation of vitamins can disrupt microbe–host interactions by altering microbial vitamin competition and sharing [48].

## Vitamin biosynthesis as a drug target for new antibiotics

Vitamin biosynthetic pathways can be interesting drug targets as humans do not possess the enzymes for de novo synthesis while many microorganisms do. Therefore, host toxicity can be limited, which is especially interesting for pathogenic fungi where toxicity is a recurrent problem due to the high resemblance of pathways and subcellular structures, as fungi and humans are both eukaryotes. Especially the riboflavin (vitamin B_2_), pantothenic acid (vitamin B_5_), and folate (vitamin B_9_) biosynthesis pathways seem to be interesting drug targets as they are well conserved among fungi and essential for fungal growth [49]. Furthermore, vitamins are precursors for cofactors that are involved in a wide variety of metabolic processes. Their absence will trigger a wide inhibitory cascade of many metabolic processes. Additionally, the substrates and products of these enzymes are well known and have a high potential to be druggable [49].

Due to these promising aspects, researchers have investigated these pathways to inhibit microbes. Recently, roseoflavin, a riboflavin analogue, was found to inhibit the proliferation of the malaria parasite [50]. Also, riboflavin synthase was selected as a therapeutic target to inhibit gut microbes involved in the development of colorectal cancer [51].

An important factor to keep into account when targeting vitamin synthesis is the presence of the vitamins in the human gut. Some pathogens can take up external vitamins from the gut lumen, which can circumvent the drug’s mode of action.

Overall, microbial vitamin biosynthesis, and its effect on the host and commensal species, highlight the importance of the microbiota in supplying micronutrients. However, further insides regarding the molecular mechanisms of cross-talk between the microbiota and the human host are necessary to gain a deeper understanding of its role in human health and for the development of new therapeutic strategies.

## References

[1] Hooper LV, Wong MH, Thelin A, Hansson L, Falk PG, Gordon JI. Molecular analysis of commensal host-microbial relationships in the intestine. Science. 2001;291(5505):881–884. Epub 2001/02/07. doi: 10.1126/science.291.5505.881 . 11157169

[2] Geuking MB, Cahenzli J, Lawson MA, Ng DC, Slack E, Hapfelmeier S, et al. Intestinal bacterial colonization induces mutualistic regulatory T cell responses. Immunity. 2011;34(5):794–806. Epub 2011/05/21. doi: 10.1016/j.immuni.2011.03.021 . 21596591

[3] Niu W, Yang F, Fu Z, Dong Y, Zhang Z, Ju J. The role of enteric dysbacteriosis and modulation of gut microbiota in the treatment of inflammatory bowel disease. Microb Pathog. 2022;165:105381. Epub 2022/01/03. doi: 10.1016/j.micpath.2021.105381 . 34974123

[4] Mosegaard S, Dipace G, Bross P, Carlsen J, Gregersen N, Olsen RKJ. Riboflavin Deficiency-Implications for General Human Health and Inborn Errors of Metabolism. Int J Mol Sci. 2020;21(11). Epub 2020/06/03. doi: 10.3390/ijms21113847 ; PubMed Central PMCID: PMC7312377. 32481712 PMC7312377

[5] Hill MJ. Intestinal flora and endogenous vitamin synthesis. Eur J Cancer Prev. 1997;6:S43–S45. Epub 1997/03/01. doi: 10.1097/00008469-199703001-00009 . 9167138

[6] Frick PG, Riedler G, Brögli H. Dose response and minimal daily requirement for vitamin K in man. J Appl Physiol. 1967;23(3):387–389. Epub 1967/09/01. doi: 10.1152/jappl.1967.23.3.387 . 6047959

[7] LeBlanc JG, Milani C, de Giori GS, Sesma F, van Sinderen D, Ventura M. Bacteria as vitamin suppliers to their host: a gut microbiota perspective. Curr Opin Biotechnol. 2013;24(2):160–168. Epub 2012/09/04. doi: 10.1016/j.copbio.2012.08.005 . 22940212

[8] Magnúsdóttir S, Ravcheev D, de Crécy-Lagard V, Thiele I. Systematic genome assessment of B-vitamin biosynthesis suggests co-operation among gut microbes. Front Genet. 2015:6. doi: 10.3389/fgene.2015.00148 25941533 PMC4403557

[9] Pham VT, Fehlbaum S, Seifert N, Richard N, Bruins MJ, Sybesma W, et al. Effects of colon-targeted vitamins on the composition and metabolic activity of the human gut microbiome- a pilot study. Gut Microbes. 2021;13(1):1–20. Epub 2021/02/23. doi: 10.1080/19490976.2021.1875774 ; PubMed Central PMCID: PMC7899684. 33615992 PMC7899684

[10] Shi Y, Cao Q, Sun J, Hu X, Su Z, Xu Y, et al. The opportunistic pathogen *Pseudomonas aeruginosa* exploits bacterial biotin synthesis pathway to benefit its infectivity. PLoS Pathog. 2023;19(1):e1011110. Epub 2023/01/24. doi: 10.1371/journal.ppat.1011110 ; PubMed Central PMCID: PMC9894557. 36689471 PMC9894557

[11] Demuyser L, Palmans I, Vandecruys P, Van Dijck P. Molecular Elucidation of Riboflavin Production and Regulation in *Candida albicans*, toward a Novel Antifungal Drug Target. mSphere. 2020;5(4). Epub 2020/08/08. doi: 10.1128/mSphere.00714-20 ; PubMed Central PMCID: PMC7407072. 32759338 PMC7407072

[12] Al Tanoury Z, Piskunov A, Rochette-Egly C. Vitamin A and retinoid signaling: genomic and nongenomic effects. J Lipid Res. 2013;54(7):1761–1775. Epub 2013/02/27. doi: 10.1194/jlr.R030833 ; PubMed Central PMCID: PMC3679380. 23440512 PMC3679380

[13] Bonakdar M, Czuba LC, Han G, Zhong G, Luong H, Isoherranen N, et al. Gut commensals expand vitamin A metabolic capacity of the mammalian host. Cell Host Microbe. 2022;30(8):1084–1092 e5. Epub 2022/07/22. doi: 10.1016/j.chom.2022.06.011 ; PubMed Central PMCID: PMC9378501. 35863343 PMC9378501

[14] Jovic TH, Ali SR, Ibrahim N, Jessop ZM, Tarassoli SP, Dobbs TD, et al. Could Vitamins Help in the Fight Against COVID-19? Nutrients. 2020;12(9). Epub 2020/08/28. doi: 10.3390/nu12092550 ; PubMed Central PMCID: PMC7551685. 32842513 PMC7551685

[15] Lei J, Xin C, Xiao W, Chen W, Song Z. The promise of endogenous and exogenous riboflavin in anti-infection. Virulence. 2021;12(1):2314–2326. Epub 2021/09/08. doi: 10.1080/21505594.2021.1963909 ; PubMed Central PMCID: PMC8425684. 34490839 PMC8425684

[16] Faust K, Raes J. Microbial interactions: from networks to models. Nat Rev Microbiol. 2012;10(8):538–550. Epub 2012/07/17. doi: 10.1038/nrmicro2832 . 22796884

[17] Franzosa EA, Sirota-Madi A, Avila-Pacheco J, Fornelos N, Haiser HJ, Reinker S, et al. Gut microbiome structure and metabolic activity in inflammatory bowel disease. Nat Microbiol. 2019;4(2):293–305. Epub 2018/12/12. doi: 10.1038/s41564-018-0306-4 ; PubMed Central PMCID: PMC6342642. 30531976 PMC6342642

[18] Sung J, Kim S, Cabatbat JJT, Jang S, Jin YS, Jung GY, et al. Global metabolic interaction network of the human gut microbiota for context-specific community-scale analysis. Nat Commun. 2017;8:15393. Epub 2017/06/07. doi: 10.1038/ncomms15393 ; PubMed Central PMCID: PMC5467172. 28585563 PMC5467172

[19] Roth JR, Lawrence JG, Bobik TA. Cobalamin (coenzyme B12): synthesis and biological significance. Annu Rev Microbiol. 1996;50:137–181. Epub 1996/01/01. doi: 10.1146/annurev.micro.50.1.137 . 8905078

[20] Shelton AN, Seth EC, Mok KC, Han AW, Jackson SN, Haft DR, et al. Uneven distribution of cobamide biosynthesis and dependence in bacteria predicted by comparative genomics. ISME J. 2019;13(3):789–804. Epub 2018/11/16. doi: 10.1038/s41396-018-0304-9 ; PubMed Central PMCID: PMC6461909. 30429574 PMC6461909

[21] Girinathan BP, DiBenedetto N, Worley JN, Peltier J, Arrieta-Ortiz ML, Immanuel SRC, et al. *In vivo* commensal control of *Clostridioides difficile* virulence. Cell Host Microbe. 2021;29(11):1693–1708 e7. Epub 2021/10/13. doi: 10.1016/j.chom.2021.09.007 ; PubMed Central PMCID: PMC8651146. 34637781 PMC8651146

[22] Brown GD, Denning DW, Gow NA, Levitz SM, Netea MG, White TC. Hidden killers: human fungal infections. Sci Transl Med. 2012;4(165):165rv13. Epub 2012/12/21. doi: 10.1126/scitranslmed.3004404 . 23253612

[23] Shao TY, Ang WXG, Jiang TT, Huang FS, Andersen H, Kinder JM, et al. Commensal *Candida albicans* Positively Calibrates Systemic Th17 Immunological Responses. Cell Host Microbe. 2019;25(3):404–417 e6. Epub 2019/03/15. doi: 10.1016/j.chom.2019.02.004 ; PubMed Central PMCID: PMC6419754. 30870622 PMC6419754

[24] Abbas CA, Sibirny AA. Genetic control of biosynthesis and transport of riboflavin and flavin nucleotides and construction of robust biotechnological producers. Microbiol Mol Biol Rev. 2011;75(2):321–360. Epub 2011/06/08. doi: 10.1128/MMBR.00030-10 ; PubMed Central PMCID: PMC3122625. 21646432 PMC3122625

[25] Doron I, Leonardi I, Li XV, Fiers WD, Semon A, Bialt-DeCelie M, et al. Human gut mycobiota tune immunity via CARD9-dependent induction of anti-fungal IgG antibodies. Cell. 2021;184(4):1017–1031 e14. Epub 2021/02/07. doi: 10.1016/j.cell.2021.01.016 ; PubMed Central PMCID: PMC7936855. 33548172 PMC7936855

[26] Tso GHW, Reales-Calderon JA, Tan ASM, Sem X, Le GTT, Tan TG, et al. Experimental evolution of a fungal pathogen into a gut symbiont. Science. 2018;362(6414):589–595. Epub 2018/11/06. doi: 10.1126/science.aat0537 . 30385579

[27] Nash AK, Auchtung TA, Wong MC, Smith DP, Gesell JR, Ross MC, et al. The gut mycobiome of the Human Microbiome Project healthy cohort. Microbiome. 2017;5(1):153. Epub 2017/11/28. doi: 10.1186/s40168-017-0373-4 ; PubMed Central PMCID: PMC5702186. 29178920 PMC5702186

[28] Klein EY, Van Boeckel TP, Martinez EM, Pant S, Gandra S, Levin SA, et al. Global increase and geographic convergence in antibiotic consumption between 2000 and 2015. Proc Natl Acad Sci U S A. 2018;115(15):E3463–E3470. Epub 2018/03/28. doi: 10.1073/pnas.1717295115 ; PubMed Central PMCID: PMC5899442. 29581252 PMC5899442

[29] Suez J, Zmora N, Segal E, Elinav E. The pros, cons, and many unknowns of probiotics. Nat Med. 2019;25(5):716–729. Epub 2019/05/08. doi: 10.1038/s41591-019-0439-x . 31061539

[30] Pandey KR, Naik SR, Vakil BV. Probiotics, prebiotics and synbiotics- a review. J Food Sci Technol. 2015;52(12):7577–7587. Epub 2015/11/26. doi: 10.1007/s13197-015-1921-1 ; PubMed Central PMCID: PMC4648921. 26604335 PMC4648921

[31] Arena MP, Russo P, Capozzi V, López P, Fiocco D, Spano G. Probiotic abilities of riboflavin-overproducing *Lactobacillus* strains: a novel promising application of probiotics. Appl Microbiol Biotechnol. 2014;98(17):7569–7581. Epub 2014/06/07. doi: 10.1007/s00253-014-5837-x . 24903812

[32] Hong DK, Yoo MS, Heo K, Shim JJ, Lee JL. Effects of *L. plantarum* HY7715 on the Gut Microbial Community and Riboflavin Production in a Three-Stage Semi-Continuous Simulated Gut System. Microorganisms. 2021;9(12). Epub 2021/12/25. doi: 10.3390/microorganisms9122478 ; PubMed Central PMCID: PMC8704370. 34946080 PMC8704370

[33] Levit R, Savoy de Giori G, de Moreno de LeBlanc A, LeBlanc JG. Protective effect of the riboflavin-overproducing strain *Lactobacillus plantarum* CRL2130 on intestinal mucositis in mice. Nutrition. 2018;54:165–172. Epub 2018/07/10. doi: 10.1016/j.nut.2018.03.056 . 29982144

[34] Boisvert WA, Mendoza I, Castañeda C, De Portocarrero L, Solomons NW, Gershoff SN, et al. Riboflavin requirement of healthy elderly humans and its relationship to macronutrient composition of the diet. J Nutr. 1993;123(5):915–925. Epub 1993/05/01. doi: 10.1093/jn/123.5.915 . 8487103

[35] Russo P, Iturria I, Mohedano ML, Caggianiello G, Rainieri S, Fiocco D, et al. Zebrafish gut colonization by mCherry-labelled lactic acid bacteria. Appl Microbiol Biotechnol. 2015;99(8):3479–3490. Epub 2015/01/15. doi: 10.1007/s00253-014-6351-x . 25586576

[36] Levit R, de Giori GS, de Moreno de LeBlanc A, LeBlanc JG. Evaluation of the effect of soymilk fermented by a riboflavin-producing *Lactobacillus plantarum* strain in a murine model of colitis. Benef Microbes. 2017;8(1):65–72. Epub 2016/11/23. doi: 10.3920/BM2016.0063 . 27873546

[37] Moyersoen I, Devleesschauwer B, Dekkers A, de Ridder K, Tafforeau J, van Camp J, et al. Intake of Fat-Soluble Vitamins in the Belgian Population: Adequacy and Contribution of Foods, Fortified Foods and Supplements. Nutrients. 2017;9(8). Epub 2017/08/12. doi: 10.3390/nu9080860 ; PubMed Central PMCID: PMC5579653. 28800115 PMC5579653

[38] Dwyer JT, Woteki C, Bailey R, Britten P, Carriquiry A, Gaine PC, et al. Fortification: new findings and implications. Nutr Rev. 2014;72(2):127–141. Epub 2014/01/23. doi: 10.1111/nure.12086 . 24447229

[39] Niedermaier T, Gredner T, Kuznia S, Schöttker B, Mons U, Lakerveld J, et al. Vitamin D food fortification in European countries: the underused potential to prevent cancer deaths. Eur J Epidemiol. 2022;37(4):309–320. Epub 2022/05/07. doi: 10.1007/s10654-022-00867-4 ; PubMed Central PMCID: PMC9187526. 35524028 PMC9187526

[40] Singh P, Rawat A, Alwakeel M, Sharif E, Al Khodor S. The potential role of vitamin D supplementation as a gut microbiota modifier in healthy individuals. Sci Rep. 2020;10(1):21641. Epub 2020/12/12. doi: 10.1038/s41598-020-77806-4 ; PubMed Central PMCID: PMC7729960. 33303854 PMC7729960

[41] Sun MG, Huang Y, Xu YH, Cao YX. Efficacy of vitamin B complex as an adjuvant therapy for the treatment of complicated vulvovaginal candidiasis: An in vivo and in vitro study. Biomed Pharmacother. 2017;88:770–777. Epub 2017/02/06. doi: 10.1016/j.biopha.2017.01.001 . 28157653

[42] Villamor E, Fawzi WW. Vitamin A supplementation: implications for morbidity and mortality in children. J Infect Dis. 2000;182(Suppl 1):S122–S133. Epub 2000/08/17. doi: 10.1086/315921 . 10944494

[43] Xie J, Zhu L, Zhu T, Jian Y, Ding Y, Zhou M, et al. Vitamin D-supplemented yogurt drink reduces *Candida* infections in a paediatric intensive care unit: a randomised, placebo-controlled clinical trial. J Hum Nutr Diet. 2019;32(4):512–517. Epub 2019/02/19. doi: 10.1111/jhn.12634 . 30773722

[44] Wierzejska RE. Dietary Supplements-For Whom? The Current State of Knowledge about the Health Effects of Selected Supplement Use. Int J Environ Res Public Health. 2021;18(17). Epub 2021/09/11. doi: 10.3390/ijerph18178897 ; PubMed Central PMCID: PMC8431076. 34501487 PMC8431076

[45] Bjelakovic G, Nikolova D, Gluud LL, Simonetti RG, Gluud C. Antioxidant supplements for prevention of mortality in healthy participants and patients with various diseases. Sao Paulo Med J. 2015;133(2):164–165. Epub 2015/05/29. doi: 10.1590/1516-3180.20151332T1 . 26018887 PMC10496628

[46] Group VTS. B vitamins in patients with recent transient ischaemic attack or stroke in the VITAmins TO Prevent Stroke (VITATOPS) trial: a randomised, double-blind, parallel, placebo-controlled trial. Lancet Neurol. 2010;9(9):855–865. Epub 2010/08/07. doi: 10.1016/S1474-4422(10)70187-3 . 20688574

[47] Anderson JJ. Oversupplementation of vitamin A and osteoporotic fractures in the elderly: to supplement or not to supplement with vitamin A. J Bone Miner Res. 2002;17(8):1359–1362. Epub 2002/08/07. doi: 10.1359/jbmr.2002.17.8.1359 . 12162488

[48] Forgie AJ, Pepin DM, Ju T, Tollenaar S, Sergi CM, Gruenheid S, et al. Over supplementation with vitamin B12 alters microbe-host interactions in the gut leading to accelerated *Citrobacter rodentium* colonization and pathogenesis in mice. Microbiome. 2023;11(1):21. Epub 2023/02/05. doi: 10.1186/s40168-023-01461-w ; PubMed Central PMCID: PMC9896722. 36737826 PMC9896722

[49] Meir Z, Osherov N. Vitamin Biosynthesis as an Antifungal Target. J Fungi (Basel). 2018;4(2). Epub 2018/06/20. doi: 10.3390/jof4020072 ; PubMed Central PMCID: PMC6023522. 29914189 PMC6023522

[50] Hemasa AL, Mack M, Saliba KJ. Roseoflavin, a Natural Riboflavin Analogue, Possesses *In Vitro* and *In Vivo* Antiplasmodial Activity. Antimicrob Agents Chemother. 2022;66(10):e0054022. Epub 2022/09/13. doi: 10.1128/aac.00540-22 ; PubMed Central PMCID: PMC9578400. 36094195 PMC9578400

[51] Alturki NA, Mashraqi MM, Jalal K, Khan K, Basharat Z, Alzamami A. Therapeutic Target Identification and Inhibitor Screening against Riboflavin Synthase of Colorectal Cancer Associated *Fusobacterium nucleatum*. Cancers (Basel). 2022;14(24). Epub 2022/12/24. doi: 10.3390/cancers14246260 ; PubMed Central PMCID: PMC9777469. 36551744 PMC9777469

